# Investigation of high flow nasal cannule efficiency with electric impedance tomography based parameters in COVID-19 adults patients: a retrospective study

**DOI:** 10.7717/peerj.15555

**Published:** 2023-07-14

**Authors:** Özlem Öner, Begum Ergan, Ayse Sezin Kizil, Mehmet Cagatay Gurkok, Esra Dugral, Necati Gökmen

**Affiliations:** 1 Faculty of Medicine Department of Anesthesiology and Reanimation, Subdivision of Critical Care Medicine, Dokuz Eylül University, İzmir, Turkey; 2 University Faculty of Medicine Department of Pulmonary, Subdivision of Critical Care, Dokuz Eylül University, İzmir, Turkey; 3 Faculty of Medicine Department of General Surgery, Subdivision of Critical Care Medicine, Dokuz Eylül University, İzmir, Turkey; 4 Pulmonologist and Physiology Specialist, İzmir Katip Çelebi Research and Training Hospital, İzmir, Turkey

**Keywords:** Covid-19, High flow nasal cannule, Efficiency, Electric impedence tomography

## Abstract

**Background/Aim:**

This study aimed to investigate the effects of oxygen therapy using a high flow nasal cannula (HFNC) on patients diagnosed with COVID-19 Acute Respiratory Distress Syndrome (C-ARDS) by utilizing electrical impedance tomography (EIT)-based parameters.

**Materials and Methods:**

Oxygen therapy was administered to the patients at two different flow rates and two different positions: T0—baseline measurements were taken in the supine position before any therapy was initiated. T1—HFNC was administered in the supine position with a flow rate of 30 L/min. T2—HFNC was administered in the supine position with a flow rate of 50 L/min. T3—HFNC was administered in the prone position with a flow rate of 30 L/min. T4—HFNC was administered in the prone position with a flow rate of 50 L/min. EIT-based parameters (global inhomogeneity index (GI index), center of ventilation (CoV), regional ventilation delay index (RVD index), region of interest ratio (ROI ratio)), as well as respiratory and hemodynamic parameters of the patients, were recorded from the database.

**Results:**

A total of twenty patients were included in this retrospective observational study. The mean age of the included patients was 64.3 ± 10.6 years. Statistically significant differences were observed in the measurements of GI index, CoV, RVD index, ROI ratio, PaO2/FiO2 ratio, respiratory rate, and mean arterial pressure parameters across different time intervals (*p* < 0.005). Pairwise comparisons of EIT parameters and measurements of respiratory and hemodynamic parameters at five different time points revealed statistically significant differences. For the GI index, significant differences were observed between the mean measurements taken at T0–T1, T0–T2, T0–T3, T0–T4, T1–T3, T1–T4, T2–T3, T2–T4, and T3–T4 time intervals (*p* < 0.05). Regarding CoV, significant differences were found between the mean measurements taken at T0–T3, T1–T3, T2–T3, and T3–T4 time intervals (*p* < 0.05). Additionally, for the ROI ratio, significant differences were observed between the measurement averages taken at each time interval (*p* < 0.05).

**Conclusion:**

Our findings suggest that prone positioning during the management of C-ARDS patients leads to improved lung homogeneity, as indicated by EIT parameters. However, further research is required to enhance the visualization of ventilation using EIT.

## Introduction

Respiratory failure is one of the main reasons for admission of COVID-19 patients to the intensive care unit (ICU) (Perier et al., 2020). Acute Respiratory Distress Syndrome (C-ARDS), which develops due to COVID-19, is a specific syndrome although it can meet the Berlin criteria (Ferguson et al., 2012). Its main features are the inconsistency of the severity of hypoxemia with respiratory mechanics and the heterogeneity of the ventilation feature (Gattinoni et al., 2020b).

Gattinoni et al. (2020a), defined two types of the clinical picture in C-ARDS cases. In type 1, in addition to near-normal pulmonary compliance, the lung fields that can be recruited are low, related to hypoxemia, hypoxic pulmonary vasoconstriction, and impaired pulm onary perfusion (Marini & Gattinoni, 2020). Therefore, high PEEP on a mechanical ventilator and prone position (PP) does not improve oxygenation in type 1 C-ARDS patients (Goligher, Ranieri & Slutsky, 2021). On the other hand, in type 2 C-ARDS, there is decreased pulmonary compliance and less recruitable lung areas (Chiumello et al., 2020). In the clinical picture of these patients, there is severe hypoxemia and consequently increased inspiratory effort and high respiratory rate (Goligher, Ranieri & Slutsky, 2021). Type 2 C-ARDS responds to a high PEEP, recruitment manoeuvre and prone position in the mechanical ventilator. High values of dynamic lung strain (lung deformation caused by tidal volume) and static lung strain (lung deformation caused by positive end-expiratory pressure (PEEP)) are associated with lung injury (González-López et al., 2012; Terragni et al., 2007). However, little is known about the effect of flow rates followed in spontaneous breathing and applied in HFNC treatment on lungs fields (Zhang et al., 2020). The main challenge here is to find the individualised optimum flow rate in HFNC therapy, just as the optimal PEEP for each patient in invasive mechanical ventilation therapy. We hypothesized that we could evaluate personalized HFNC flow rate and PP improved ventilation using EIT parameters.

Oxygen therapy with high-flow nasal cannula (HFNC) is a non-invasive and good option when hypoxia develops, but intubation indications do not occur in patients with C-ARDS (Li et al., 2021). During the COVID-19 pandemic, HFNC therapy has received significant attention as a bridging therapy function before and after intubation in the ICU (Zhang et al., 2020). HFNC may be a valuable and viable treatment option for patients with C-ARDS with remarkable clinical advantages (Crimi et al., 2022). However, the application of high gas flows is initially unclear as to the effect on the lung tissue, as well as the aerosolization of the droplets, the transmission of infection and doubts about the safety of the device. Another method of improving gas exchange in ARDS patients is to follow the patient in the prone position (PP) (Gattinoni et al., 2019). However, the response to PP in C-ARDS patients is unpredictable and may differ in each patient (Coppo et al., 2020).

Different phenotypes of C-ARDS require the development of patient-specific personalized ventilation strategies (Goligher, Ranieri & Slutsky, 2021). This is only possible by simultaneous monitoring of the effects of invasive or non-invasive mechanical ventilation therapy applied to the patient with devices such as bedside electrical impedance tomography (EIT) (Mauri et al., 2017). Although computed tomography (CT) is a valuable tool for imaging patients with C-ARDS, its routine use in COVID-19 patients is limited due to radiation load, cost, difficulties in referral of critically ill patients, and low vital signs. in the intensive care unit, unstable in the CT room and risk of contamination for healthcare workers (Perier et al., 2020).

We hypothesized that we could evaluate personalized HFNC flow rate and PV improved ventilation using EIT parameters. In our study, in patients followed in the intensive care unit with the diagnosis of C-ARDS, at two different flow rates and in two different positions; It was aimed to investigate the effect of oxygen therapy with HFNC with EIT-based parameters. According to the authors’ literature, although there are studies examining HFNC efficacy with EIT parameters, no studies have been identified in the prone position and in adults COVID-19 patients (Scaramuzzo et al., 2022).

## Materials and Methods

### Patient selection

This study was planned after approval from the University Ethics Committee (approval no: 2022/05-01) as a retrospective review of the hospital data of 20 concecutive patients who were diagnosed with COVID-19 confirmed by polymerase chain reaction (PCR) test, followed in the COVID ICU, and diagnosed with ARDS according to the Berlin criteria (Table 1) (Ferguson et al., 2012).The informed written consent form was obtained from the patients. Posterior-anterior chest X-rays and thorax computed tomography images of the patients were consistent with the typical infiltration and ground glass images of COVID-19 ARDS.

**Table 1:** Main characteristics of the study population.

Patients	Age	F/M	APACHEII	BMI	ROX INDEX	Days of intubation	Days of mortality	Days of discharge	MALIGNITY Yes/NO	DM YES/NO	HT YES/NO	CRF YES/NO	CAD YES/NO	ARF YES/NO
1	79	M	22	31	4.46	5	9		1	0	0	0	0	0
2	53	M	5	37	5.89	–	–	12	0	1	1	0	0	0
3	66	F	24	31	6.01	–	–	14	0	1	1	0	0	0
4	59	M	15	27	5.72	–	–	4	0	0	1	0	0	0
5	60	F	8	28	6.67	–	–	4	0	0	1	0	0	0
6	70	M	12	26	5.69	–	–	1	0	1	1	0	0	0
7	72	M	14	28	5.96	–	–	6	0	0	1	1	0	0
8	68	M	19	32	6.72	–	–	10	0	0	1	0	0	0
9	75	F	26	27	4.25	6	10	–	0	1	1	0	1	0
10	66	M	16	30	6.56	–	–	9	0	0	1	0	0	1
11	54	M	16	26	7.38	–	–	6	0	1	0	0	0	0
12	39	F	14	34	4.47	–	–	8	0	0	1	0	0	0
13	65	F	21	24	4.37	2	12	–	0	0	0	1	1	0
14	68	M	11	21	6.23	–	–	5	0	0	1	0	0	0
15	57	M	9	23	6.41	–	–	8	0	1	1	0	0	1
16	49	F	18	25	4.36	7	13	–	0	0	1	1	0	1
17	61	M	14	26	4.48	–	–	11	0	1	1	0	0	0
18	76	M	16	31	4.78	3	9	–	0	1	0	1	0	0
19	67	F	22	32	4.95	2	5	–	0	1	1	1	0	0
20	82	M	24	23	4.87	1	3	–	0	1	1	0	0	0
Summary	64.3 ± 10.6	7/13	16.3 ± 5.7	28.1 ± 4.1	5.5 ± 0.9	3.7 ± 2.2	8.7 ± 3.6	7.5 ± 3.6	1/19	10/10	16/4	5/15	2/18	3/17

**Notes.**

Abbreviation F female M Male APACHE II Acute physiology and chronic health evaluation BMI Body Mass İndex ROX index SpO_2_/FiO_2_ ratio to respiratory rate DM Diabetes Mellitus HT Hipertansion CRF Chronic Renal Failure CAD Coronary Artery Disease ARF Acute Renal Failure

Exclusion criteria from the study were accepted as being younger than 18 years of age, being pregnant and lactating, having a body mass index (BMI) above 50 kg/m^2,^ having a rib cage malformation, presence of pneumothorax, and conditions where EIT monitoring is contraindicated (patients with automatic implantable cardioversion defibrillator, automatic drug pumps, patients with chest skin injury (Kunst et al., 1999).

The patients, which followed for COVID-19 ARDS and administered respiratory support with HFNC, demographic data, body mass indexes (BMI), APACHE-II score (acute physiology and chronic health evaluation), ratios of PaO_2_ (partial oxygen pressure) in blood gas at baseline and during treatment, to FiO_2_ (percentage of oxygen delivered in HFNC) (PaO_2_/FiO_2_ ratios), respiratory rate (RR), peripheral oxygen saturation (SpO_2_), ROX index (SpO_2_/FiO_2_ ratio to RR) and hemodynamic parameters, including heart rate (HR) and mean arterial pressure (MAP), position information during therapy (supine-prone), flow values applied in HFNC, and EIT images were obtained retrospectively from the recordings. All of the blood gases taken from the patients were taken at least 20 min after the treatment in order to reflect the position and flow changes in the blood gas parameters. In addition, if intubation was performed, the days of intubation, the days of discharge from ICU, and mortality rates were retrospectively scanned from the data registration form.

All the patients in our research group were given all medical treatments (Favipiravir, corticosteroids, anticoagulant and if necessary, anabiotic *etc*.) in accordance with the National Covid Treatment Guideline published by the Ministry of Health of the Republic of Turkey (Kurulu, 2021).

### Study protocol

In the study protocol, the flow rate of the HFNC was increased to two preset levels (30 L/min and 50 L/min), and each flow level was maintained for 20 min to a minimum. The reason for holding a minimum of 20 min in each position is that this is the minimum time required for any changes to be reflected in blood gas measurements. As a part of routine care in our clinic, arterial blood gas measurement is performed 20 min after each change. Because it is common practice to wait 20 to 30 min after the FiO_2_ is changed before arterial blood gas measurements are obtained, 10 min may be adequate unless the patient has obstructive lung disease which requires a longer equilibration time (Hess et al., 2016). For this retrospective study, data of patients admitted to ICU with respiratory failure due to COVID-19 were obtained from the hospital data system and records on the EIT. In our clinic, this patient group is followed in two different flow rates (30 L/min and 50 L/min) and two different positions as part of routine care. Patients undergo lung imaging with EIT during HFNC treatment. In order to find the optimum flow rate during the treatment, arterial blood gas is taken at least 20 min after the flow level and position change. We think that it should take 20–30 min for the change to be reflected in the arterial blood gas.

Oxygen levels (FiO_2_) in the HFNC were adjusted so that the saturation levels of the patients would not decrease from 90–92% (for patients with Type 2 respiratory failure, the saturation would not fall below 88–90%).

As invasive mechanical ventilation indications: include level of consciousness (Glasgow coma score <12), cardiac arrest/arrhythmias, severe hemodynamic instability (norepinephrine>0.1 µg/kg/min and persistent or worsening respiratory status and lack of oxygenation (despite HFNC flow ≥ 50 L/min and FiO_2_>100; PaO_2_<60 mmHg), respiratory acidosis (PaCO_2_>50 mmHg, pH <7.25), respiratory rate >30 bpm or inability to clear secretions, and a ROX index below 2.85 were accepted (Mauri et al., 2017).

For all patients followed up, the temperature was set to 37 °C with the HFNC (Optiflow, Fisher & Paykel Healthcare, Auckland, New Zealand) humidifier (MR850, Fisher & Paykel Healthcare, Auckland, New Zealand), and oxygen was administered nasally with a medium silicone nasal cannula (RT050/051, Fisher & Paykel Healthcare, Auckland, New Zealand). Heat and humidified high flow nasal cannula or as most call it, hi flow nasal cannula (HFNC), is not just a standard nasal cannula cranked up to very high flow rates. It actually takes gas and can heat it to 37 °C with a 100% relative humidity and can deliver 0.21–1.00% fi02 at flow rates of up to 60 liters/min. The flow rate and FiO2 can be independently titrated based on the patient’s flow and fi02 requirements (Sharma et al., 2023). The patients were asked to breathe through the nose, and their mouths and noses were covered with a simple surgical mask due to the risk of contamination. The different flow rates and position information in the study were recorded as follows:

T0: Baseline measurements are taken in the supine position before any therapy is started.

T1: HFNC in supine position with a flow rate of 30 L/min.

T2: HFNC in supine position with a flow rate of 50 L/min.

T3: HFNC in prone position with a flow rate of 30 L/min.

T4: HFNC in prone position with a flow rate of 50 L/min.

In routine practice in our clinic, all patients admitted to the ICU are primarily treated in the supine position. However, in diseases such as C-ARDS where oxygenation does not improve in the supine position, patients are placed in the prone position to correct ventilation-perfusion mismatch. Therefore, the patients admitted to the ICU were first placed in the supine position and then in the prone position. For this reason, our research also accepted the supine position as the baseline.

### EIT measurements

EIT measurements were performed on the Pulmovista 500 device (Dräger Medical, Lübeck, Germany), which is part of routine care in our clinic. In our clinic, instead of lung imaging with CT, imaging with EIT is applied to patients followed up with C-ARDS. For application, the silicone EIT belt with 16 surface electrodes was placed around the patients’ rib cage in the fourth intercostal space and then connected to the EIT monitor for visualisation. The reflections from tissues were measured by applying alternative current to lungs and surrounding tissue. The stimulation frequency and amplitude were adjusted automatically by the EIT device. EIT measurements were performed continuously at 20 Hz (Tomasino et al., 2020). When the patients were turned to the prone position, attention was paid to tying the EIT belt in the same spots by placing marker points so that the position of the EIT belt would not change. In addition, the data is digitally filtered with a cut-off frequency of 0.67 Hz to avoid reflection of heart-related impedance changes (Tomasino et al., 2020). EIT scans consist of 32 × 32 colour-coded matrix impedance images (Tomasino et al., 2020).

### EIT data

In the data obtained with EIT, lung regions are divided into four regions of interest (ROI): by recording regional changes of waveforms resulting from ventilation, non-dependant ROI1 and ROI2 show right and left ventral lung regions; ROI3 and ROI 4 show dependent right and left dorsal lung areas (Zhang et al., 2020). In the supine position, both lung portions of the patient in the ventral region are defined as non-dependant, and both lung portions in the dorsal region are defined as dependent regions. While ventilation is high in non-dependant lung regions, perfusion is high in dorsal lung areas defined as dependant. It is defined as ventilation-perfusion mismatch, especially in ARDS patients. Similar to this definition, ROI areas showing ventral lung areas are defined as non-dependant, while ROI areas showing dorsal lung areas are defined as dependant. The total ventilation of the four ROI areas is 100% and approximately 25% in each ROI regions (Bickenbach et al., 2017). The ROI ratio (also called ROI ratio or impedance ratio) divides the ventilation activity of the ventral region by the dorsal ventilation activity of the fEIT images, and may indicate both lung recruitment and derecruitment (Tomasino et al., 2020). An ROI ratio close to 1 was accepted as indicating a homogeneous distribution of ventilation (Zhao et al., 2009).

Dräger PulmoVista 500 device is used in our clinic. In the Pulmovista 500 device, the lung is divided into 4 ROI (horizontal or quadrants) areas. A region of interest (ROI) is a user-defined area within a status image. The image can be divided into four ROIs and arranged either horizontally, as quadrants or in a customised way. The area covered by each ROI is represented by the corresponding regional impedance waveform and the regional numeric value (Teschner, Imhoff & Leonhardt, 2015).

ROI ratio is the ratio between the mean values of ventral (ROI 1 and ROI 2) and dorsal areas (ROI 3 and ROI 4) and represents a reliable measures of the ventilation distribution (Tomasino et al., 2020). Although the ROI ratio value close to 1 is accepted as an indicator of homogeneity, in another recent study, this ratio was accepted to be between 0.6 and 1.5 (Tomasino et al., 2020; Yang et al., 2021). However, in our study, we took the ROI ratio as 1 as a reference when evaluating the data.

The present study examined the proportional changes of ROI areas in different flows and positions, and the homogeneity of ventilation was determined. Image reconstruction was performed using the algorithm in the software EIT Data Review Tool (Dräger Medical, Lübeck, Germany). EIT data were analysed offline using MATLAB R2015 (The MathWorks, Inc., Natick, MA, USA) as programmed software.

### EIT data analysis

To reduce the heterogeneity of spontaneous breathing when analysing EIT data, an analysis of 5-minute continuous EIT data (data averaged) was performed at five time points (T0, T1, T2, T3, T4) (Kunst et al., 1999). Three parameters were examined to analyse the EIT data of patients treated with HFNC in spontaneous respiration using the software program. In addition to the software program used, it is possible to obtain the relevant parameters with the help of the formulas given below.

The GI index was introduced to quantify tidal volum distribution within the lungs and aims to summarize the complex pulmonary impedance distribution pattern using a single numeric value (Eq. (1)) (Putensen et al., 2019). The ideal value for healthy individuals is 0.5 (Bickenbach et al., 2017). Increased GI index is in parallel with lung injury (Zhao et al., 2009). It has been used to set the optimum positive end-expiratory pressure (PEEP) value in studies (Putensen et al., 2019). The lowest possible GI index corresponds to the optimum PEEP level (Putensen et al., 2019).

(1) \begin{eqnarray*}GI= \frac{\sum _{X{Y}_{\in \mathrm{lung}}}{|}{\mathrm{DI}}_{XY}-\mathrm{Median}({\mathrm{DI}}_{\mathrm{lung}}){|}}{\sum _{X{Y}_{\in \mathrm{lung}}}{\mathrm{DI}}_{XY}} \end{eqnarray*}

DI = differential impedance, DI_xy_ = a defined pixel lung region, and DI_lung_ = all pixels represented in lungs (Zhao et al., 2009).

The regional ventilation delay index (RVD index) was developed to further analyse regional ventilation (Eq. (2)) (Muders et al., 2012; Wrigge et al., 2008). It expresses the time and delays from the beginning of inspiration to the opening of the closed alveoli (Putensen et al., 2019). Although the RVD index was initially developed for slow flow manoeuvres during invasive mechanical ventilation, it was later used for spontaneous breathing studies (Bickenbach et al., 2017). To avoid confusion, the RVD in spontaneous breathing studies was named RVD_sponbreath_ (Bickenbach et al., 2017). The value presented in our study is the RVD _sponbreathvalue_.

(2) \begin{eqnarray*}RV{D}_{\dot {I}}= \frac{{t}_{i,40\text{%}}}{{T}_{inspiration,global}} \times 100\text{%}\end{eqnarray*}

t_*i*40%_ indicates the time required to reach 40% of the maximum inspiratory impedance change, and T_inspiration,global_ indicates the expiration time calculated from the global impedance curve (Muders et al., 2012).

Center of ventilation (CoV) is a measure that defines the spatial distribution of ventilation (Putensen et al., 2019). It was defined as the weighted average of the geometric centres of lungs ventilation in the dorsal-ventral and right-left direction (eqn-(3)) (Frerichs et al., 1998). A CoV value of 50% indicates that the ventilation distribution is centred in the dorsal-ventral direction of the thorax (Putensen et al., 2019). A decrease in CoV values indicates that ventilation shifts towards non-dependent ventral lung regions due to the collapse of alveoli in dependent dorsal lung regions (Putensen et al., 2019).

(3) \begin{eqnarray*}CoV= \frac{\sum ({y}_{i}\times T{V}_{i})}{\sum T{V}_{i}} \times 100\end{eqnarray*}

TV _i_ represents the impedance change in EIT images, and the other value, Y_i_, is the pixel height scaled dorsal and ventrally (Frerichs et al., 1998).

## Statistical Analysis

Parametric tests were used without the normality test due to the compatibility of the Central Limit Theorem (Norman, 2010). In the data analysis, the mean and standard deviation were used when making the statistics of the continuous data, and the frequency and percentage values were used when defining the categorical variables. Repeated ANOVA test statistic was used to compare the means of more than two dependent groups. In case of difference between the means in more than two repeated measurements, pairwise comparisons were evaluated with the Bonferroni statistic. The statistical significance level of the data was taken as *p* < 0.05. In evaluating the data, (http://www.e-picos.com), New York Biostatistics software and MedCalc statistical package program were used .

## Results

The mean age of 20 patients who met the study’s inclusion criteria was 64.3 ± 10.6 years. Thirteen of the patients were male, and seven were female. The mean APACHE II score calculated in the first 24 h of admission to the ICU was 16.3 ± 5.7, and the mean ROX indices calculated before starting the treatment were 5.5 ± 0.9. The detailed descriptive characteristics of the patients are summarised in Table 1 (pages 10–11).

At T0 (Baseline measurements are taken in the supine position before any therapy is started)-T4(HFNC in prone position with a flow rate of 50 L/min) time points ; EIT parameters, respiratory and hemodynamic parameters effects of HFNC therapy:

When T0 points (baseline measurements are taken in the supine position before any therapy is started) and T4 points (HFNC in prone position with a flow rate of 50 L/min) are compared; GI index, CoV, RVDsponbreating index, ROI ratio, MAP, RR decrease and P/F ratio increase were found to be statistically significant (Table 2) (page 11).

**Table 2:** Difference statistics in repeated measurements of EIT parameters, respiratory and hemodynamic parameters (*n* = 20).

	**T1**	**T2**	**T3**	**T4**	**T5**	**p value**
Variables	$\bar {X}\pm $ SD	$\bar {X}\pm $ SD	$\bar {X}\pm $ SD	$\bar {X}\pm $ SD	$\bar {X}\pm $ SD	
GI INDEX	0.68 ± 0.03	0.59 ± 0.02	0.61 ± 0.02	0.5 ± 0.01	0.55 ± 0.02	<0.0001
COV	42.64 ± 1.84	44.53 ± 2.01	43.7 ± 1.05	50.67 ± 1.33	43.98 ± 0.93	<0.0001
P/F ratio	104.8 ± 15.23	123.3 ± 22.95	130 ± 23.61	147.85 ± 28.35	203.1 ± 33.44	<0.0001
RR	40.95 ± 3.69	33.25 ± 3.69	31.05 ± 3.43	26.3 ± 3.19	21.3 ± 3.57	<0.0001
MAP	90.3 ± 7.88	87.85 ± 12.65	81.35 ± 14.74	78.25 ± 10.35	81.5 ± 10.95	0.004
RVD_sponbreath_ index	4.21 ± 0.5	4.11 ± 0.74	4.24 ± 0.59	4.08 ± 0.71	3.38 ± 1.13	0.006
ROI ratio	2.005 ± 0.14	1.83 ± 0.22	1.68 ± 0.23	1.11 ± 0.08	1.39 ± 0.16	<0.0001

**Notes.**

Abbreviation GI Index Global Homogeneity Index Cov Center of Ventilation RVD_sponbreath_ index Regional Ventilation Delay Index ROI Ratio ROI1+ROI2/ROI3+ROI4 P/F Ratio PaO_2_/FiO _2_ Ratio RR Respiratory Rate MAP Mean Arterial Pressure

### As a result of pairwise comparison of repeated measurements of EIT parameters, respiratory and hemodynamic parameters

Table 3 shows a significant difference between repeated measurements of the measurement parameter in terms of GI index (*p* < 0.05). Among the five measurements made for the GI index, It was observed that the difference between the mean measurements taken at T0–T1, T0–T2, T0–T3, T0–T4, T1–T3, T1–T4, T2–T3, T2–T4 and T3-T4 time intervals was statistically significant (*p* < 0.05). On the other hand, GI index measurement at T1–T2 time points was not found to be significant (*p* = 0.06)(Table 3; pages 12, 13,14).

**Table 3:** Pairwise comparison statistics for repeated measurements of EIT parameters, respiratory and hemodynamic parameters and traits (*n* = 20).

	**Mean difference**	*p* value	**95% Confidence interval for difference**	
**Time**			**Lower bound**	**Upper bound**
**GI index**				
T1–T2	.09^*^	<0.0001	0.07	0.11
T1–T3	0.07^*^	<0.0001	0.04	0.09
T1–T4	0.18^*^	<0.0001	0.16	0.2
T1–T5	0.13^*^	<0.0001	0.09	0.15
T2–T3	−0.21	0.06	−0.04	0.001
T2–T4	0.09^*^	<0.0001	0.08	0.11
T2–T5	0.04^*^	<0.0001	0.02	0.06
T3–T4	0.12^*^	<0.0001	0.09	0.14
T3–T5	0.06^*^	<0.0001	0.04	0.09
T4–T5	−0.05^*^	<0.0001	−0.07	−0.04
**CoV**				
T1–T2	−0.89	0.99	−2.78	0.99
T1–T3	−0.06	0.99	−1.78	1.66
T1–T4	−7.02^*^	<0.0001	−8.45	−5.6
T1–T5	−0.34	0.99	−1.69	1.01
T2–T3	0.83	0.99	−0.81	2.47
T2–T4	−6.13^*^	<0.0001	−7.98	−4.29
T2–T5	0.54	0.99	−0.91	1.99
T3–T4	−6.96^*^	<0.0001	−8.02	−5.91
T3–T5	−0.28	0.99	−1.42	0.85
T4–T5	6.68^*^	<0.0001	5.46	7.9
**P/F ratio**				
T1–T2	−18.5^*^	0.01	−33.84	−3.16
T1–T3	−25.2^*^	<0.0001	−39.91	−10.49
T1–T4	−43.05^*^	<0.0001	−61.14	−24.96
T1–T5	−98.3^*^	<0.0001	−122.25	−74.35
T2–T3	−6.7^*^	0.03	−13.06	−0.34
T2–T4	−24.55^*^	<0.0001	−32.57	−16.53
T2–T5	−79.8^*^	<0.0001	−100.67	−58.93
T3–T4	−17.85^*^	<0.0001	−26.81	−8.89
T3–T5	−73.1^*^	<0.0001	−95.35	−50.84
T4–T5	−55.25^*^	<0.0001	−76.45	−34.05
**RR**				
T1–T2	7.7^*^	<0.0001	5.25	10.15
T1–T3	9.9^*^	<0.0001	6.74	13.06
T1–T4	14.65^*^	<0.0001	11.88	17.42
T1–T5	19.65^*^	<0.0001	16.16	23.14
T2–T3	2.2^*^	0.04	0.09	4.31
T2–T4	6.95^*^	<0.0001	5.09	8.81
T2–T5	11.95^*^	<0.0001	9.68	14.22
T3–T4	4.75^*^	<0.0001	3.58	5.92
T3–T5	9.75^*^	<0.0001	8.43	11.07
T4–T5	5^*^	<0.0001	3.72	6.28
**MAP**				
T1–T2	2.45	0.99	−9.09	13.99
T1–T3	8.95	0.32	−3.3	21.2
T1–T4	12.05^*^	<0.0001	4.81	19.29
T1–T5	8.8	0.11	−1.08	18.67
T2–T3	6.5	0.12	−0.87	13.87
T2–T4	9.6	0.08	−0.66	19.86
T2–T5	6.35	0.87	−4.79	17.49
T3–T4	3.1	0.99	−5.81	12.01
T3–T5	−0.15	0.99	−10.8	10.5
T4–T5	−3.25	0.99	−12.98	6.49
**RVD**_**sponbreath**_ **index**				
T1–T2	0.09	0.99	−0.57	0.75
T1–T3	−0.3	0.99	−0.61	0.54
T1–T4	0.13	0.99	−0.5	0.76
T1–T5	0.83	0.11	−0.09	1.76
T2–T3	−0.12	0.99	−0.84	0.59
T2–T4	0.04	0.99	−0.64	0.73
T2–T5	0.74	0.44	−0.35	1.82
T3–T4	0.16	0.99	−0.49	0.81
T3–T5	0.86^*^	0.052	0.006	1.73
T4–T5	0.69	0.43	−0.32	1.72
**ROI ratio**				
T1–T2	0.18^*^	<0.0001	0.08	0.28
T1–T3	0.32^*^	<0.0001	0.22	0.43
T1–T4	0.9^*^	<0.0001	0.79	1.008
T1–T5	0.61^*^	<0.0001	0.52	0.7
T2–T3	0.15^*^	<0.0001	0.06	0.24
T2–T4	0.72^*^	<0.0001	0.57	0.88
T2–T5	0.44^*^	<0.0001	0.33	0.54
T3–T4	0.58^*^	<0.0001	0.42	0.73
T3–T5	0.29^*^	<0.0001	0.19	0.38
T4–T5	−0.29^*^	<0.0001	−0.38	−0.19

**Notes.**

Abbreviation GI Index Global Homogeneity Index Cov Center of ventilation RVD_sponbreath_ index Regional ventilation delay index ROI ratio ROI1+ROI2/ROI3+ROI4 P/F ratio PaO_2_/FiO _2_ Ratio RR Respiratory rate MAP Mean arterial pressure

* The mean difference is significant at the 0.05 level. Adjustment for multiple comparisons: Bonferro.

Table 3 shows a significant difference between repeated measurements of the measurement parameter in terms of CoV (*p* < 0.05). Among the five measurements made for CoV, the difference between the mean measurements taken at T0–T3, T1–T3, T2–T3 and T3–T4 time intervals was statistically significant (*p* < 0.05). However CoV measurements at T0 and T1, T2, and T4 time points were not significant (*p* = 0.99). In addition, CoV measurements at T1 and T2–T4 time points and T2–T4 time points were not found significant (*p* = 0.99). (Table 3; pages 12, 13,14)

Table 3 shows a significant difference between repeated measurements of the RVD index measurement parameter (*p* < 0.05). Among the five measurements made for RDV, the difference between the mean measurements taken at T2–T4 time intervals was found to be statistically significant (Table 3; pages 12, 13,14) (*p* < 0.05).

Table 3 shows a significant difference between repeated measurements of the ROI ratio measurement parameter (*p* < 0.05). Among the five measurements made for ROI ratio, it was observed that the difference between the measurement averages taken at each time interval was statistically significant (Table 3; pages 12, 13, 14)) (*p* < 0.05).

Table 3 shows a significant difference between repeated measurements of the P/F ratio measurement parameter (*p* < 0.05). Among the five measurements made for the P/F ratio, it was seen that the difference between the measurement averages taken at all time intervals was statistically significant (Table 3; pages 12, 13, 14) (*p* < 0.05).

Table 3 shows a significant difference between repeated measurements of the RR measurement parameter (*p* < 0.05). It was observed that the difference between the means of measurements taken at all time intervals from the five measurements made for RR was statistically significant (Table 3; pages 12, 13, 14) (*p* < 0.05).

Table 3 shows a significant difference between repeated measurements of the MAP measurement parameter (*p* < 0.05). Among the five measurements for MAP, the difference between the mean measurements taken at T0-T3 time intervals was found to be statistically significant (*p* < 0.05). In addition, there was no significant difference in MAP measurements between T0 time points and T1, T2, T4 time points, T1 time points and T2, T3, T4 time points, and T2 and T3, T4 time points (Table 3; pages 12, 13, 14).

The change in evolution of estimated marginal means of GI index, CoV, RVD_sponbreathvalue_, P/F ratio, respiratory rate, mean arterial pressure and ROI ratio are provided in Figs. 1–8. EIT images is reconstruction by MATLAB in Fig. 9.

## Discussion

In the present study, HFNC efficiency was investigated in COVID-19 patients using EIT parameters, respiratory and hemodynamic parameters. We found a decrease in GI index, CoV, RVDindex, ROI ratio, respiratory rate and mean arterial pressure, and an increase in P/F ratio in PV when the flow rate of HFNC was increased from 30 L/min to 50 L/min.

We found that GI index and CoV values decreased at 30 L/min flow rate in PP and HFNC. Similarly, when evaluated according to the ROI ratio, the lung heterogeneity decreased in the prone position and at low flow rate. A statistically significant decrease was found in the RVD_sponbreathindex_ between the supine position and the PP at HFNC 50 L/min flow rate.

**Figure 1:**
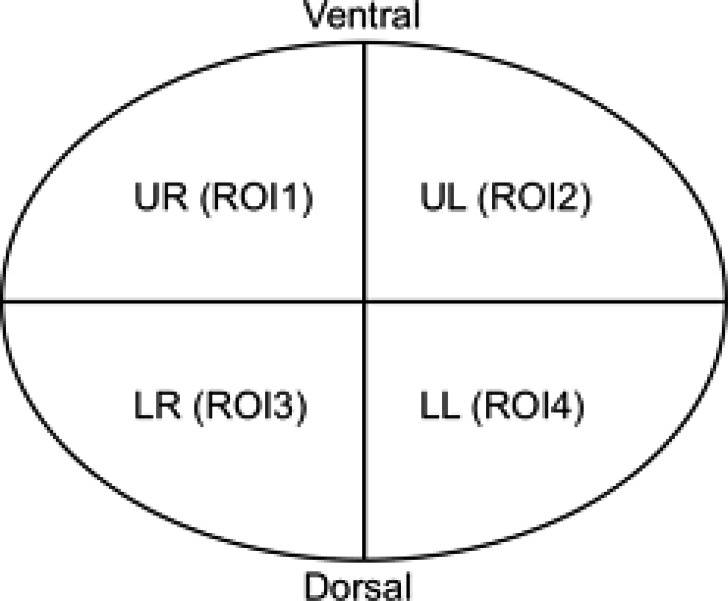
Non-dependant (ROI 1-ROI 2) and dependent (ROI 3-ROI 4) ROI areas in the supine position.

**Figure 2:**
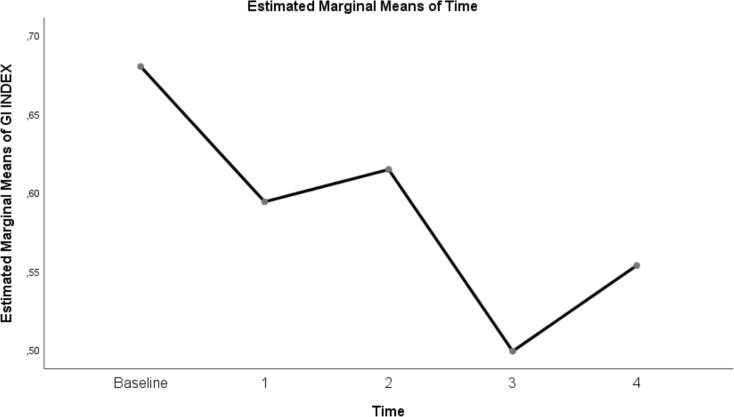
Evolution of estimated marginal means of GI INDEX at different times.

**Figure 3:**
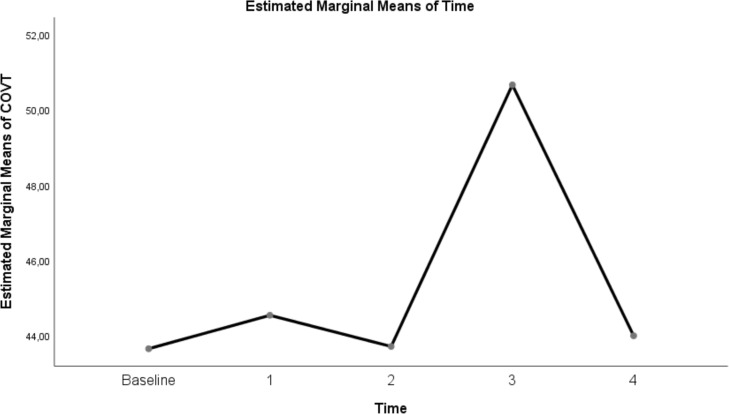
Evolution of estimated marginal means of CoV at different times.

**Figure 4:**
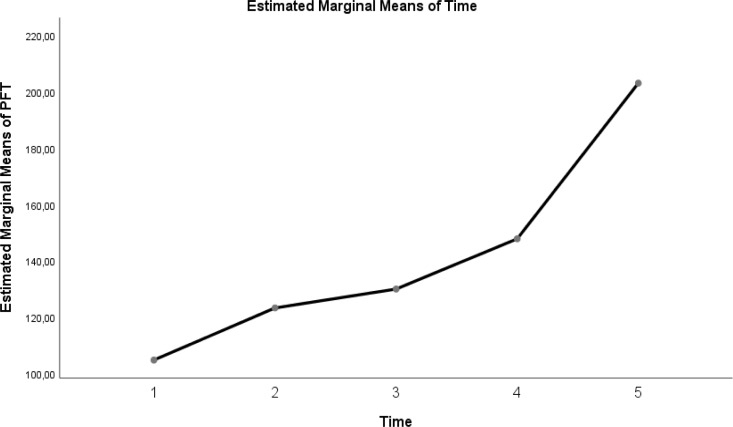
Evolution of estimated marginal means of PF at different times.

**Figure 5:**
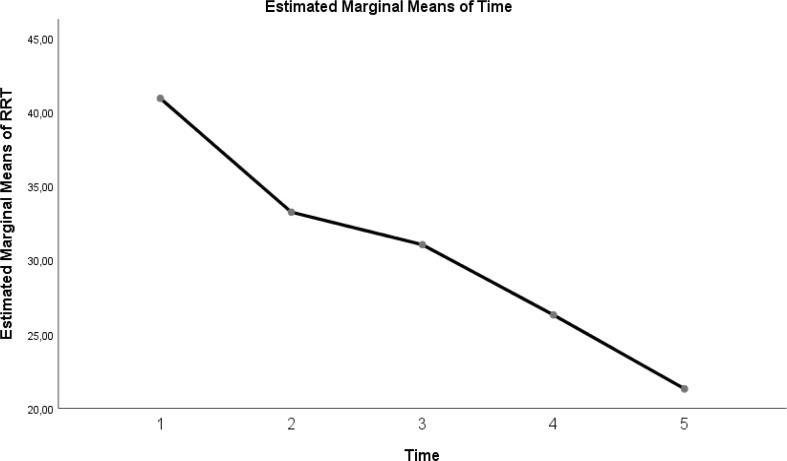
Evolution of estimated marginal means of RR at different times.

**Figure 6:**
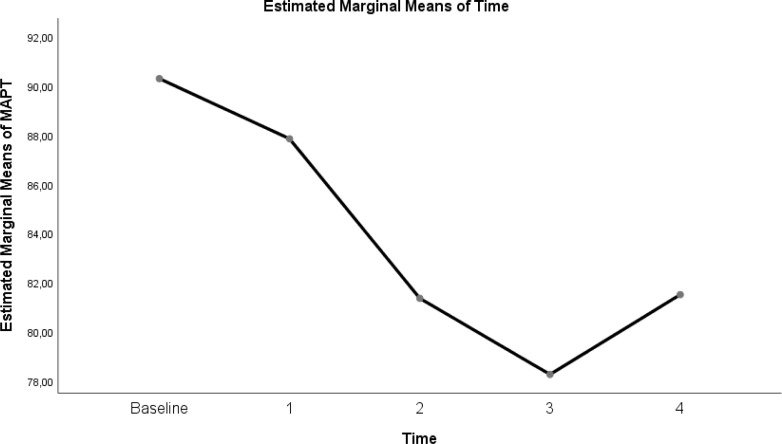
Evolution of estimated marginal means of MAP at different times.

**Figure 7:**
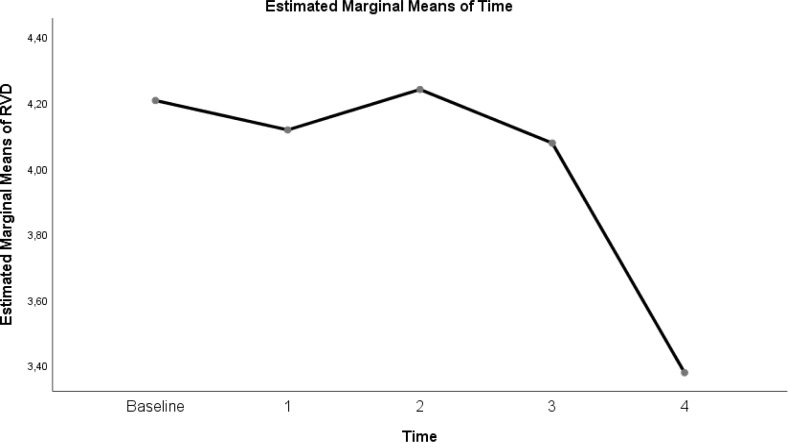
Evolution of estimated marginal means of RVD at different times.

**Figure 8:**
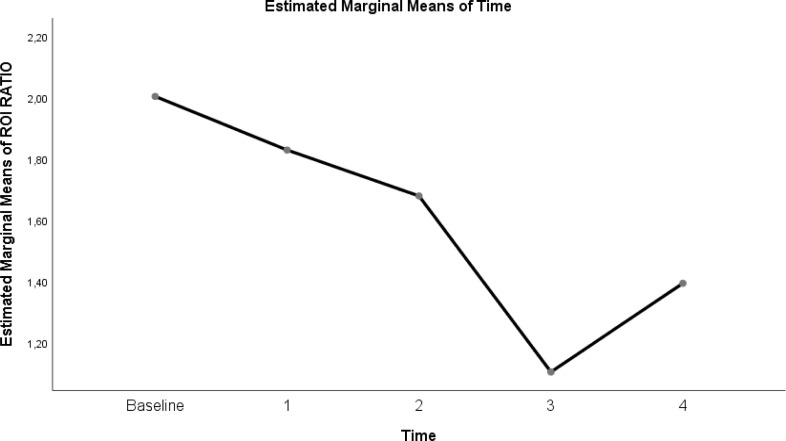
Evolution of estimated marginal means of ROI RATIO at different times.

HFNC reduces inspiratory effort, improves lung mechanics, and reduces minute volume by affecting PEEP (Bräunlich et al., 2013). HFNC flow rates are heterogeneous, ranging from 15 L/min to 100 L/min (Hernández et al., 2016). To evaluate the effects of different HFNC flow rates on lungs, EIT-based and most commonly used GI index, CoV, RVD index, and ROI ratio parameters were used in this study (Putensen et al., 2019). These parameters are EIT-based parameters used to describe not only the effects of HFNC therapy on the lung, but also the effects of invasive and non-invasive mechanical ventilation therapy on the lungs (Putensen et al., 2019).

**Figure 9:**
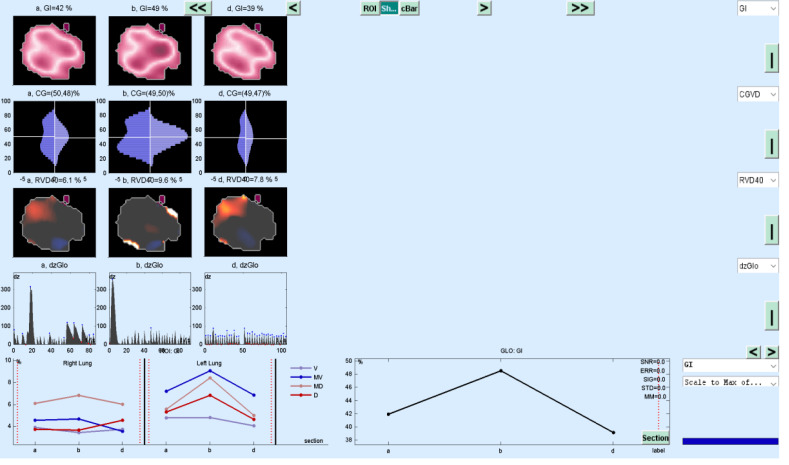
EIT image reconstruction with MATLAB.

The GI index is a functional EIT parameter widely used to measure the heterogeneity of lung ventilation (Putensen et al., 2019). When the GI index values in our study were examined, we found that ventilation was homogeneously distributed at 30 L/min and 50 L/min flow rates in the prone position compared to baseline values before treatment. We found that the GI index at a flow rate of 30 L/min in the prone position was 0.5 ± 0.01 and almost homogeneous. This result shows that it may be beneficial to increase the flow rate in HFNC by titration in case of improvement in P/F ratios and a decrease in respiratory rate.

On the other hand, although it was determined that the GI index decreased and homogeneity increased at 30 L/min and 50 L/min flow rates in the supine position compared to the pre-treatment values, there was no statistically significant difference between this position and these flow rates. Similarly, in Li et al. (2021) study, no statistically significant difference was found in the GI index in the measurements they made at different flow rates after HFNC treatment was started in the supine position. It was found that the difference in our study was due to the fact that the patients were followed in the prone position, and therefore, the GI index level decreased, and homogeneity increased in the prone position compared to the supine position. As in ARDS patients, the rationale for following the patient in the prone position in C-ARDS patients is to ensure that the atelectatic areas in the dependent dorsal region are included in ventilation and to reduce heterogeneous ventilation (Guérin et al., 2020).

At the same time, the prone position reduces the ventilation/perfusion mismatch, hypoxemia, and shunt, which is typical of ARDS (Perier et al., 2020). Because when the patient is in the prone position, the pleural pressure gradient between the dependent and non-dependent region decreases as a result of gravity; thus, a more homogeneous lung is formed (Munshi et al., 2017).

Another EIT parameter that measures ventilation distribution is CoV. When the CoV values in this study were examined, the CoV value at a flow rate of 30 L/min in the prone position was 50.67 ± 1.33. This value indicates that the ventilation distribution is centred in the dorsal-ventral direction of the thorax (Kunst et al., 1999). CoV values were decreased at HFNC supine 30 L/min, 50 L/min and prone 50 L/min flow rates. This can be explained by the shift of ventilation from the dorsal lung areas to the ventral lung fields in ARDS patients, resulting in increased aeration and even overdistension in the ventral region while decreasing and causing atelectasis in the dorsal regions (Bachmann et al., 2018; Wang et al., 2022).

Similarly, Li et al. (2021), in their study, found that ventilation was reduced in the dorsal regions of the lung in patients in whom HFNC treatment was unsuccessful. This resulted in increased respiratory effort, shortened inspiratory time, decreased minute volume, and inadequate oxygenation in the patients’ group. Similarly, in our study, respiratory rate and P/F ratios were found to be higher and P/F ratios lower in the supine position, where the CoV value was low and heterogeneous lung characteristics persisted, compared to the prone position.

In our study of the other EIT parameter, the RVD _sponbreath_ index, a significant difference was found only between 50 L/min in the supine position and 50 L/min in the prone. This can be explained by the inspiratory time being too short during spontaneous breathing to calculate a fixed RVD index (Yang et al., 2021). Bickenbach et al. (2017) found the RVD_sponbreath_ index to be statistically significant in their study of spontaneous breathing trials in prolonged weaning (Yang et al., 2021). However, RVD_sponbreath_ is strongly dependent on the individual variation, depth, and respiratory rate of the patient’s spontaneous breathing (Kunst et al., 1999). Therefore, when calculating the RVD_sponbreath_ value, a sequence with spontaneous respirations should be selected (Bickenbach et al., 2017; Kunst et al., 1999). Although a 5-minute sequence was chosen to reduce the heterogeneity of spontaneous breathing, only the RVD_sponbreath_ between the two times was statistically significant. As a result, RVD, which was initially developed to measure slow flow in mechanical ventilators, is used during spontaneous breathing, but it is incomprehensible among patients (Wrigge et al., 2008).

It is a cheap, simple and effective method to follow the patient in the prone position in ARDS patients and has a 15% benefit on absolute survival (Dalla Corte et al., 2020). It also significantly improves arterial oxygenation compared to the supine position (Guérin et al., 2013). In the study we presented, significant improvements were found in P/F ratios, RR, and MAP in patients placed in the prone position. In previous studies, there are findings that a prone position can reduce ventilator-related lung damage and improve respiratory and hemodynamic parameters (Abroug et al., 2008). On the other hand, in a large, randomised study conducted in recent years, the P/F ratio was found to be similar between survivors and non-survivors in ARDS patients (Albert et al., 2014). The effectiveness of the prone position at the individual patient level may depend on the sub-phenotypes of ARDS, as in C-ARDS (Famous et al., 2017).

Another parameter developed to monitor the physiological effects of EIT, which is the dynamic monitoring of regional lung mechanics at the bedside, is the ROI ratio (Putensen et al., 2019). The value obtained by the ratio of the dependent ROI areas to the non-depending ROI areas approached the value of 1 at a flow rate of 30 L/min in the prone position in our study. This shows the homogeneity of lung ventilation. In the COVID-19 ARDS case report presented by Tomasino et al. (2020), while the patient with Gattinoni type I ARDS had an ROI ratio of 1 in the supine position, the value decreased in the prone position, overdistension occurred, and oxygenation did not improve. More studies are needed to predict the ROI ratio, ARDS subtype, and future treatment.

### Limitation

Our study has all the limitations of a retrospective study. Also, limited equipment and personnel were employed during the pandemic. In our study, in which the effects of HFNC application with EIT have monitored in patients followed up with the diagnosis of C-ARDS, the sample size was limited. However, the sample size could not be calculated since there has been no previous study on this subject in the literature. It is necessary to carry out studies with larger sample groups in the future. Further studies are needed in the future for the effect of EIT in visualizing ventilation.

### Conclusion

The COVID-19 pandemic still remains a cause for global concern. EIT is a non-invasive, radiation-free clinical imaging tool. EIT has the potential to monitor the effectiveness of the prone position and to determine flow rates in HFNC treatment in COVID-19 ARDS patients. Positioning the patient, which can simply be summarised as “keep the healthy lung down”, is an effective treatment option to improve ventilation. According to the authors’ literature knowledge, our study is promising because it is the first study in the world to examine HFNC treatment in COVID-19 patients with EIT and the first EIT study in our country.

##  Supplemental Information

10.7717/peerj.15555/supp-1 Supplemental Information 1 Data

## Additional Information and Declarations

### Data Availability

The following information was supplied regarding data availability: The raw data is in the Supplementary File.
